# Nature’s chemical computer: Herbal residue-derived carbon dots build logic gates for H_2_O_2_ tracking and ecological antimicrobials

**DOI:** 10.1016/j.mtbio.2025.102574

**Published:** 2025-11-20

**Authors:** Xiangru Hou, Denggerile Ao, Lu Ga, Gang Dai, Jun Ai

**Affiliations:** a College of Chemistry and Enviromental Science, Inner Mongolia Key Laboratory of Environmental Chemistry, Inner Mongolia Normal University, 81 Zhaowudalu, Hohhot, 010022, China; b College of Pharmacy, Inner Mongolia Medical University, Jinchuankaifaqu, Hohhot, 010110, China

**Keywords:** Fe-CDs, Logic gate, Antibacterial, Colorimetric, Peroxidase-like activity

## Abstract

In this work, we report the use of logic platforms and iron-doped carbon dots (Fe-CDs) as a promising peroxidase-like analogue for the qualitative quantification of hydrogen peroxide (H_2_O_2_). The synthesised Fe-CDs exhibited excellent peroxidase-like (POD-like) activity compared to carbon dots (CDs), which catalyzed the formation of blue oxidation product (ox-TMB) with maximum absorption at 652 nm from the chromogenic substrate 3,3′, 5,5′ - tetramethylbenzidine (TMB). Steady state kinetic analysis shows that Fe-CDs have strong POD-like activity. Using the POD-like activity of Fe-CDs, H_2_O_2_ can be specifically detected colorimetrically. Based on this, we constructed a logic platform for H_2_O_2_ colourimetric sensing using Fe-CDs and H_2_O_2_ as inputs and the colorimetric signal as output. The H_2_O_2_ concentration and absorbance values showed good linearity in the range of 60–800 μM (R^2^ = 0.9887) with a limit of detection (LOD) of 7.25 μM, and the current method was successfully applied to the detection in mouthwash samples and laboratory tap water with recoveries in the range of 96.39 %–104.01 %. Fe-CDs catalyse the production of reactive oxygen species from H_2_O_2_, which can kill both Gram-positive and Gram-negative bacteria, and Fe-CDs have a highly efficient antimicrobial effect compared to CDs. Fe-CDs can be as high as 99.89 % antibacterial against *E. coli* and 97 % against *S. aureus*. In conclusion, we used herbal dregs to prepare Fe-CDs antimicrobial agent, and by recycling herbal dregs, the resources were maximised, and the nanozymes can not only construct the logical platform for H_2_O_2_ colorimetric sensing, but also show good application prospects in antimicrobials.

## Graphical abstract

**Figure:**
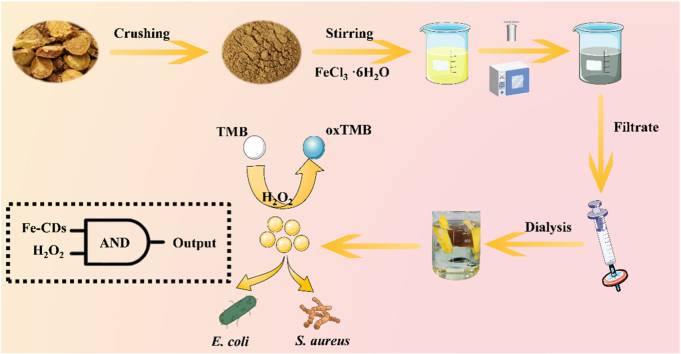

## 1 Introduction

Over the past decade or so, researchers have worked to develop artificial enzymes to replace natural enzymes, and with advances in nanotechnology, nanozymes have caught the attention of researchers. Nanozymes are highly efficient nanomaterials with properties similar to those of natural enzymes, which have the advantages of high stability, adjustable catalytic activity, low preparation cost, and long storage time, which can effectively avoid the inherent defects of natural enzymes, and show good application prospects in many fields of biomedicine, environmental treatment, agriculture and food [1]. Miao et al. [2] reported a photoperforation technique that utilizes iron oxide photothermal nanoparticles embedded in electrospun nanofibers to deliver payload molecules to cells while avoiding direct contact between cells and nanoparticles. This strategy avoids the potential long-term toxicity of nanoparticles and helps to utilize photoperforated cells for therapeutic purposes. To date, a variety of nanozymes have been developed, including noble metals [3], metal oxides [4], carbon-based nanomaterials [5], and metal-organic frameworks (MOFs) [6]. Among them, carbon-based nanomaterials with enzyme-like activity have become new candidates in the field of materials. Carbon atoms can be bonded in various ways to produce isomers with different properties, such as graphene [7], carbon dots (CDs) [8], carbon nanotubes (CNTs) [9], fullerenes [10], and amorphous carbon [11], which are all carbon isomers with peroxidase-like (POD-like) activity.

CDs have become a research hotspot due to their excellent water dispersion, high stability, high resistance to photobleaching and easy surface modification [12]. CDs are zero-dimensional nanomaterials with a particle size of less than 10 nm. Compared with graphene oxide and CNTs, CDs have a larger specific surface area and more surface functional groups, which gives them the potential to incorporate heteroatoms [13], and the modification of the CDs with a variety of metallic or non-metallic atoms can significantly improve the chemical properties. Shi et al. [14] initially found POD-like activity in some CDs, which were chromogenic in the presence of hydrogen peroxide (H_2_O_2_) in response to the peroxidase substrate 3,3′,5,5′-tetramethylbenzidine (TMB), which was oxidised to form blue oxidised TMB (oxTMB). Du et al. [15] prepared Si-CDs from peanut shells by a one-pot hydrothermal method, which exhibited POD-like activity and could be used for the detection of cysteine (Cys) in foods. Various heteroatom-doped CDs have been reported to exhibit enhanced POD-like activity due to doping with non-metallic or metallic elements that can change the valence state [16,17]. Compared to non-metals, metal atoms have more empty orbitals and thus are a better kind of electron donor, and doping of metal atoms can induce new properties in CDs, resulting in the generation of new energy levels in the bandgap of CDs and facilitating charge transfer in CDs [18]. In recent years, various metal doped CDs have been developed to simulate peroxidase activity. Among them, comparative studies have shown that Fe-CDs often exhibit better catalytic performance than other metal doped CDs due to their unique coordination environment and electronic structure [19,20]. Based on this, this study selects Fe-CDs as the research object, aiming to further explore their wide applications. The logic gates are devices that can perform Boolean logic operations and are the basic components of integrated circuits used for information processing and storage. It uses “1” for “high signal” and “0” for “low signal”. In recent years, the development of logic gates at the molecular level has received a lot of attention, and the rapid development of molecular logic gates has advanced the development of logic computation using biomolecules as the basic building blocks [21]. de Silva’s team [22] reported for the first time AND molecular logic gate with H^+^ and Na^+^ as inputs and fluorescence as output. Typically, logic operations are performed by one or more inputs and produce measurable output signals, and the most commonly used logic operating platforms today consist of input signals capable of triggering a change in the fluorescence signal and a fluorescence output signal [21]. Combining nanozymes with logic gates is a novel and attractive research direction, and the cross-application of the two brings new opportunities and ideas for various fields, widely used in research in environmental monitoring, disease diagnosis and treatment, food safety testing, biomedical and other fields [23]. In 2019, Wang’s team [24] designed a dual amplification cascaded logic gate AND circuit controlled by dual miRNAs for detecting cancer cells. Bhat et al. [25] constructed an INHIBIT gate with F^−^ and AcO^−^ ions as inputs and absorbance as output, which can be visually observed by colorimetric method. The changes in absorbance can be used to identify ion content, and the operation is simple with good development prospects. The POD-like activity of nanozyme is used to catalyse the oxidation of TMB to generate blue products, and the colorimetric signal is used as the output, so that the phenomenon can be observed with the naked eye without complicated instruments, which is suitable for rapid on-site screening.

Herbal medicine is an important part of Chinese traditional medicine, which has gradually gained attention worldwide, and its unique theoretical system and clinical efficacy contribute to human health [26]. However, China produces about 70 million tonnes of herbal residues annually, and most of the herbal residues have low recycling rates, leading to a huge waste of resources [27]. Currently, most of the treatment methods for herbal residues are stacking, landfilling or incineration, but all of them lead to serious environmental pollution and resource waste [28]. Therefore, it is necessary to develop these herbal residues into environmentally friendly and useable materials to reduce environmental pollution and maximise the use of resources to achieve the sustainable effect of “waste for waste”. Herbal residues are rich in cellulose, hemicellulose, lignin and various trace elements [29], which can be used to synthesise CDs, reducing the potential toxicity associated with the synthesis of chemicals. Moreover, the synthesis of derived CDs from herbal pomace has the advantages of simple preparation method, abundant raw materials, low cost as well as low toxicity, which makes it an ideal precursor material [30]. Scutellaria baicalensis has been reported [31] to be widely used due to its good anti-inflammatory and antibacterial effects, and its main components are compounds such as flavonoids, terpenoids, and polysaccharides. Among them, flavonoids have a certain inhibitory ability to bacteria, fungi and polysaccharides can be used by microorganisms to accelerate the decomposition of substances. Therefore, we employed the common herb Scutellaria baicalensis and used its dregs to synthesise derived CDs to further explore their POD-like activity and antibacterial effects.

Bacterial infections are a serious threat to human health, triggering cell, tissue, and organ dysfunction or death, which can lead to serious diseases and complications [32]. Antibiotics remain the mainstay of treatment for bacterial infections, but bacteria are increasingly resistant to antibiotics and there is an urgent need to develop a safe and effective way to kill bacteria. Currently regarding antimicrobial alternatives are phages, antimicrobial peptides and CDs [[33], [34], [35]]. However, the chemical instability of phage and antimicrobial peptides as well as the complex production process hinder further practical applications [36], making CDs a promising alternative to antibiotics in comparison.

In this work, we synthesised Fe-CDs and CDs by a one-step hydrothermal method using Scutellaria baicalensis dregs as a precursor material, and Fe-CDs possessed significantly enhanced POD-like activity compared to CDs. In the presence of H_2_O_2_, Fe-CDs catalyse the oxidation of TMB to produce the blue product oxTMB, thus allowing specific detection of H_2_O_2_. Based on this principle, we constructed the AND logic platform for colourimetric sensing of H_2_O_2_ and verified the feasibility in real samples. Fe-CDs with POD-like properties can catalyse the conversion of H_2_O_2_ into highly oxidative ·OH, which can cause oxidative damage to bacteria, attacking the bacterial cell membrane and leading to bacterial death. These findings not only provide a new direction for further research and development of nanozymes, but also promote the application and development of nanomaterials in biomedicine and tumour therapy.

## 2 Reagents and instruments

### 2.1 Reagents

Ferric chloride hexahydrate (FeCl_3_·6H_2_O) was purchased from Beijing mreda Technology Co., Ltd; 3,3′, 5,5′ - tetramethylbenzidine (TMB) was purchased from Beijing Solarbio Science & Technology Co., Ltd; Hydrogen peroxide (H_2_O_2_) and sodium acetate buffer solution (HAc-NaAc) were purchased from Aladdin Reagent Co., Ltd; Sodium chloride (NaCl) was purchased from Sinopharm Chemical Reagent Co., Ltd; Absolute ethanol (CH_3_CH_2_OH, ≥99.7 %) was purchased from Tianjin Zhiyuan Chemical Reagent Co., Ltd; Hydrochloric acid (HCl) was purchased from Shanghai Macklin Biochemical Co., Ltd. The water used during the experiments was ultrapure water (18.20 MQ·cm) prepared by Milli. Q ultrapure water system. Scutellaria baicalensis herbal residue is the waste residue left after extracting the active substance of the drug, which is obtained after drying in air.

### 2.2 Instruments

The experimental instruments used in the experiments were as follows, F97pro fluorescence spectrometer (Shanghai Prismatic Technology Corporation), U-2900 UV–Vis Spectrometer (Hitachi High-Technologies Corporation), JEOL-2100F transmission electron microscope (Jeol Corporation, Japan), EQ-100DE CNC Ultrasonic Cleaner (Kunshan Ultrasonic Instrument Corporation), ESCALAB 250Xi X-ray photoelectron spectroscopy (Thermo Scientific, USA), Nicolet iS50 fourier transform infrared spectrometer (Thermo Fisher Scientific, USA), X-ray diffractometer (Japanese Regaku Ultima Type IV), D1008 high speed centrifuge (Beijing Dalong Xingchuang Experimental Instrument Co., Ltd.), Guohua multi head magnetic heating stirrer (Changzhou Instrument Manufacturing Co., Ltd.), Hitachi S-4800 energy dispersive X-ray spectroscopy, ZQZY-78BN oscillating incubator (Shanghai Zhichu Instrument Co., Ltd.), HR1500--Ⅱ A2(KY) biosafety cabinet (Qingdao Haier biomedical Co., Ltd.), SX-500 steam sterilizer (TOMY KOGYO CO.,LTO), SPX-150B-Z biochemical incubator (Shanghai Boxun medical biological Instrument Co., Ltd.), DHG-9015A blast drying oven (Shanghai Yiheng Scientific Instrument Co., Ltd.), ST210 pH electrode (aohaosi Instrument Co., Ltd., USA).

## 3 Experimental section

### 3.1 Synthesis of CDs

The synthesis of this CDs was obtained by a slightly modified preparation based on the synthetic method of Xie et al. [37]. Weighed 3.0 g of Scutellaria baicalensis dregs powder dissolved in 30 ml of ultrapure water, mixed homogeneously and transferred to a teflon-lined autoclave, the reaction was carried out at 200 °C for 3 h. At the end of the reaction, the reactor was left to cool down naturally to room temperature, and centrifugation was carried out at 10,000 rpm for 5 min in order to remove impurities and interfering substances. The supernatant was taken and the initial product was filtered using a 0.22 μm microporous filter membrane and then dialysed in ultrapure water using a dialysis bag (molecular retention = 1000) for 6 h. It was stored in a refrigerator at 4 °C for subsequent use.

### 3.2 Synthesis of Fe-CDs

Fe-CDs were synthesised by a one-step hydrothermal method with FeCl_3_·6H_2_O as the iron source. Briefly, 3.0 g of Scutellaria baicalensis dregs powder was weighed and dissolved in 30 ml of ultrapure water, and 1 g of FeCl_3_·6H_2_O was added dropwise gradually while stirring, which was mixed well and then transferred to a teflon-lined autoclave, and then reacted at 200 °C for 4 h. At the end of the reaction, the reactor was left to cool naturally to room temperature, and centrifugation was performed at 10,000 rpm for 10 min in order to remove interfering substances. The supernatant was taken and the initial product was filtered using a 0.22 μm microporous filter membrane and then dialysed in ultrapure water using a dialysis bag (molecular retention = 1000) for 6 h. It was stored in a refrigerator at 4 °C for further use.

### 3.3 Studies on the activity of Fe-CDs-like peroxidases

The whole catalytic reaction was carried out in HAc-NaAc buffer solution (pH = 3.6), to which TMB (10 mM), H_2_O_2_ (30 %), Fe-CDs or CDs were added to the system, and finally the total volume was fixed by adding ultrapure water for dilution. The absorbance values of the reaction systems were subsequently recorded using a UV–Vis spectrophotometer to compare their activities.

### 3.4 Optimisation of experimental factors (pH, temperature and time)

In order to investigate the different factors affecting the peroxidase-like activity of Fe-CDs, we carried out this colourimetric reaction under different pH, temperature and time conditions. The pH was varied between 2.5 and 7.2, and the temperature was gradually increased from 4 °C to 75 °C for 5 min–30 min, and the chemical changes of TMB were monitored by UV–Vis absorption at 652 nm.

### 3.5 H_2_O_2_ sensing

Based on the fact that Fe-CDs possess strong POD-like activity, we used this analogue to catalyse the oxidation of H_2_O_2_. Firstly, different concentrations of H_2_O_2_ ranging from 60 to 800 μM were prepared. Subsequently, Fe-CDs and TMB solutions were added to the different concentrations of H_2_O_2_ solution and finally, ultrapure water was added for dilution to fix the total volume. The resulting mixture was incubated at room temperature for some time and then the UV–Visible absorbance at 652 nm was measured to monitor the change in catalytic activity.

### 3.6 Specific detection of H_2_O_2_

A solution of Fe-CDs, TMB and 1 mM common substances (D-Penicillamine (D-PA), Glucose (Glu), Fructose (Fru), Ascorbic Acid (AA), Dopamine Hydrochloride (DH), Urea, Melamine, Sucrose, and Ethylene Diamine Tetraacetic Acid (EDTA)) were each added to 2 ml centrifuge tubes, and diluted to a fixed volume by adding ultrapure water. Similarly, common metal ion solutions (Zn^2+^, Mg^2+^, Na^+^, K^+^, Fe^2+^, Mn^2+^, Cu^2+^, Co^2+^, Al^3+^, Pb^2+^) were added to the system and after incubation at room temperature, the UV–Visible absorbance at 652 nm was measured.

### 3.7 Real sample analysis

In order to explore the practical usability of the sensing system, mouthwash was purchased from a supermarket, diluted 12-fold, and then experimentally determined using the same conditions, spiking a known amount of H_2_O_2_ into the samples, assaying them using the standard curve method, and validating the results and determining the percentage recoveries using the UV–Vis absorption method. Similarly, the laboratory tap water was directly taken for the addition method to detect H_2_O_2_, and the UV–Visible absorption method was used to verify the results and determine the recovery rate.

### 3.8 Operational procedures for visual and logic gate

To perform the functions of the molecular logic gates, the two-input combination was injected into the logic system consisting of TMB (10 mM) and HAc-NaAc buffer. Fe-CDs and H_2_O_2_ (30 %) solution were used as dual inputs and each possible input was added to the logic system and the colour change of the TMB was used as the output signal to implement the operation of the AND gate. Among them, the input of Fe-CDs and H_2_O_2_ (30 %) is respectively marked as “1” and “0”, and the colorimetric signal change is respectively marked as “1” and “0”. For the input combination, Fe-CDs and H_2_O_2_ were added, and a volume of ultrapure water was added to keep the total volume of the solution constant. When the input combination is (0, 0), i.e., no Fe-CDs and H_2_O_2_ solution is added; when the input combination is (1, 0), only Fe-CDs solution is added to the system; when the input combination is (0, 1), only H_2_O_2_ solution is added to the system; and when the input combination is (1, 1), both Fe-CDs and H_2_O_2_ solutions are added to the system. Subsequently, the mixed solutions were incubated at room temperature for 25 min and the UV–Vis absorption spectra of each input were collected.

### 3.9 Bacterial culture

Luria-Bertani (LB) liquid medium was prepared by adding 10 g of peptone, 5 g of yeast extract and 5 g of sodium chloride in 1 L of ultrapure water and after mixing, it was autoclaved and the solid medium was obtained by adding 20 g of agar on top of the liquid medium. The monoclonal colonies of *Escherichia coli* (*E. coli*) and *Staphylococcus aureus* (*S. aureus*) grown on solid medium were transferred to 10 mL of liquid medium in shaking flasks and placed in a constant temperature shaking incubator for cultivation (37 °C, 120 rpm). After a period of time, the absorbance (OD_600_) was measured by a UV–Visible spectrophotometer. When the absorbance value reached 0.4–0.8, the cultures were serially diluted and spread on plates to find the appropriate concentration for subsequent experiments.

### 3.10 In vitro antibacterial assays

**Fig. 1:**
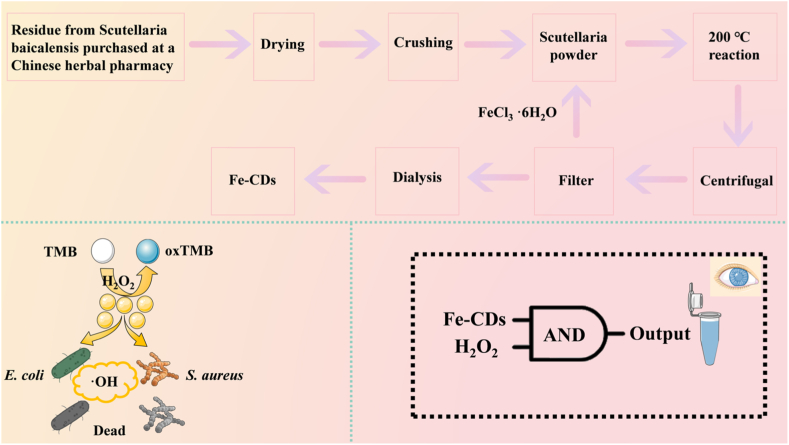
Schematic representation of colorimetric sensing and antimicrobial activity of H_2_O_2_ by modulation of peroxidase catalytic capacity of Fe-CDs.

Plate counting method was used to determine the bactericidal effect of Fe-CDs on *E. coli*, i.e., the antimicrobial performance was assessed by counting CFUs. The experiments were done in 0.9 % NaCl solution with 30 % H_2_O_2_ diluted to 1 mM. The anti *E. coli* experiments were divided into: (I) *E. coli* (II) *E. coli* + H_2_O_2_ (III) *E. coli* + Fe-CDs (IV) *E. coli* + Fe-CDs + H_2_O_2_ (V) *E. coli* + CDs + H_2_O_2_ (Ⅵ) *E. coli* + CDs. According to the above grouping, *E. coli*, Fe-CDs solution, CDs solution and H_2_O_2_ solution were added respectively, and the corresponding NaCl solution was supplemented. The final system was 1 ml. The mixed system was incubated in a constant temperature oscillation incubator (37 °C, 120 rpm) for 30 min. After half an hour, 100 μL of the mixed system was aspirated and inoculated onto LB solid medium according to the dilution-coated plate method, coated well, left to stand and placed in a biochemical incubator (37 °C), inverting the plate to prevent contamination. Photographs were taken of the petri dishes after a period of time and the growth of bacteria on each solid medium was recorded and finally the relative bacterial viability was obtained by counting the number of colonies. For *S. aureus*, the same method was used for the experiment. The inhibition rate was calculated as follows [38]:

$$\text{Antibacterial}\;\text{ratio}\;\%=\left(1-\frac{{CFU}_{\left(each\;group\right)}}{{CFU}_{\left(control\right)}}\right)\times 100\%$$

where CFU (control) is the CFU of the control group (group 1) and CFU (each group) is the CFU of the different subgroups mentioned above (see Fig. 1).

## 4 Results and discussion

### 4.1 Characterization of Fe-CDs

#### 4.1.1 Optical properties of Fe-CDs

**Fig. 2:**
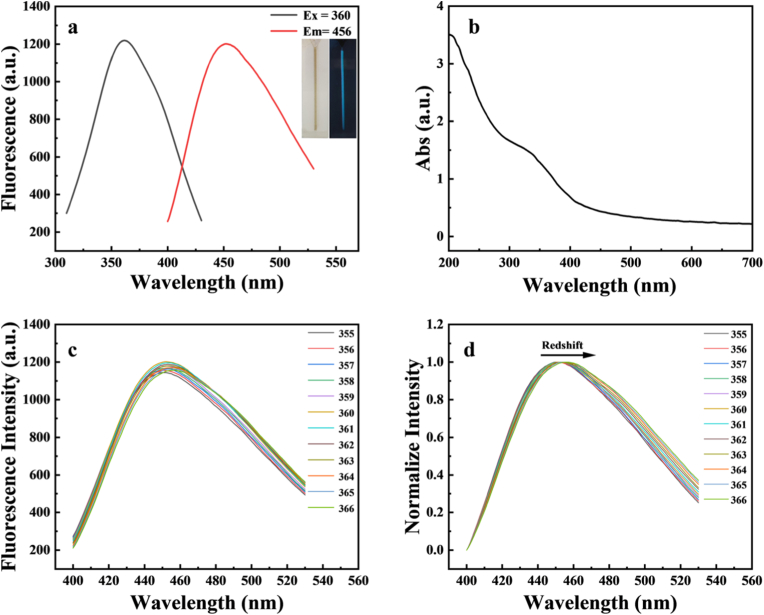
(a) Fluorescence spectra of Fe-CDs (Inset: images of Fe-CDs under natural light (left) and UV (365 nm) irradiation (right)) (b) UV–Vis absorption spectra of Fe-CDs (c) Emission spectra at different excitation wavelengths (d) Normalised spectra.

To explore the optical properties of Fe-CDs, UV absorption and detailed fluorescence studies were carried out. We prepared Fe-CDs by a one-step hydrothermal method using Scutellaria baicalensis herbal residue as a carbon source and FeCl_3_·6H_2_O as an iron source, and scanned the excitation and emission wavelengths of Fe-CDs with a fluorescence spectrophotometer. As shown in Fig. 2a, Fe-CDs showed the maximum emission wavelength at 456 nm under the excitation wavelength of 360 nm, and the solution of Fe-CDs showed light yellow colour under daylight and bright blue fluorescence under UV lamp irradiation, which was visible to the naked eye. The optimal excitation and emission wavelengths of the CDs prepared without iron doping were located at 395 nm and 493 nm, respectively, and also showed bright blue fluorescence under 365 nm UV lamp irradiation, as shown in Fig. S1a. As shown in Fig. 2b, the UV–Vis absorption spectra of Fe-CDs were tested. Fe-CDs had an absorption peak near 215 nm, which was caused by the π-π∗ transition in the conjugated system composed of C=C framework [39], and a broad absorption peak at 330 nm, which was caused by the n-π∗ transition of C=O and C=N [[40], [41], [42]]. The same was true for CDs prepared without iron doping (Fig. S1b). In addition, Fe-CDs also exhibit excitation-dependent fluorescence behavior, therefore, assessing the dependence or non-dependence of the Fe-CDs emission spectra with respect to the excitation wavelength is one of the important points that should be considered [43]. Thus, the emission spectra of Fe-CDs were evaluated at different excitation wavelength ranges from 355 to 366 nm in 11 nm increments. As shown in Fig. 2c and d, by increasing the excitation wavelength from 355 nm to 366 nm, the position and intensity of the emission wavelength have changed (the position has a red shift, and the intensity first increases and then decreases). These excitation wavelength dependent fluorescence properties of Fe-CDs can be attributed to the different sizes of nanoparticles in the sample (quantum effect) and the different functional groups on the surface of Fe-CDs [44,45]. Similarly, the emission spectra of CDs under different excitations are shown in Fig. S1c and S1d.

#### 4.1.2 Stability studies of Fe-CDs

**Fig. 3:**
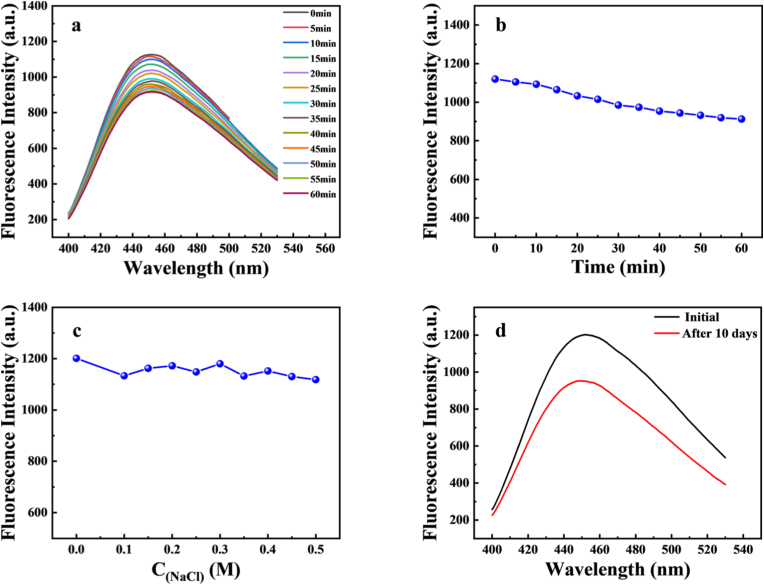
(a) Photo-bleaching fluorescence spectrogram of Fe-CDs (b) Photo-bleaching fluorescence scatter plot of Fe-CDs (c) Effect of different concentrations of NaCl solution on Fe-CDs (d) Initial fluorescence intensity of Fe-CDs vs. 10 days later fluorescence intensity of Fe-CDs.

Fig. 3 shows that the optical stability of Fe-CDs was explored under different experimental conditions. We evaluated the anti-interference and fluorescence stability of the prepared Fe-CDs by exploring the effects of different concentrations of NaCl solution, storage days and xenon lamp irradiation time on the fluorescence properties of Fe-CDs, for which the stability study is shown in Fig. S2.

The Fe-CDs were placed under xenon lamp irradiation for 1 h, as shown in Fig. 3a, and the photostability of the prepared Fe-CDs was illustrated by measuring their photobleaching properties, and the fluorescence intensities were made into a scatter plot as shown in Fig. 3b, which clearly showed that the fluorescence value was reduced by 200, indicating that the fluorescence intensity ratio of Fe-CDs was relatively stable. According to the literature [46], the photobleaching properties depend on the concentration of CDs, environment and exposure intensity. Under the irradiation of xenon lamp, the initial fluorescent molecules (which may be adsorbed on CDs) are transformed into non fluorescent forms after absorbing radiation. The chromophore slowly undergoes complete destruction, and the absorption is reduced accordingly. The fluorescence test was carried out by adding different concentrations of NaCl solution to the Fe-CDs solution, and the results are shown in Fig. 3c. When the concentration of NaCl was gradually increased to 0.5 M, its fluorescence intensity decreased to less than 100, which indicates that the Fe-CDs have strong salt tolerance, which is conducive to its application in high concentration of salt solution. To evaluate the storage stability of Fe-CDs, the effect of storage time on the strength of Fe-CDs was explored. As shown in Fig. 3d, it can be clearly seen that after 10 days, the Fe-CDs still maintain more than 80 % of the initial fluorescence, indicating that the Fe-CDs have strong resistance. In conclusion, the above results indicate that the prepared Fe-CDs exhibit excellent optical stability.

#### 4.1.3 Structural characterisation of Fe-CDs

**Fig. 4:**
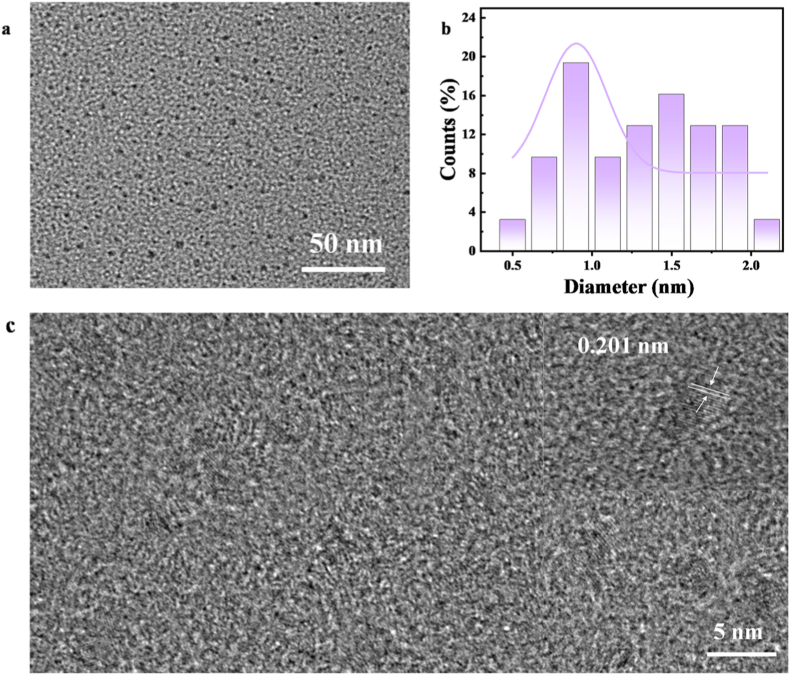
(a) TEM image of Fe-CDs (b) Histogram of particle size distribution of Fe-CDs (c) Lattice spacing of Fe-CDs.

**Fig. 5:**
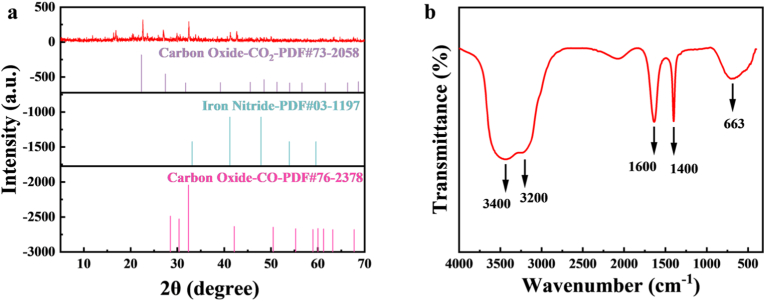
(a) XRD plot of Fe-CDs (b) FI-IR plot of Fe-CDs.

The morphology, structure, composition and surface chemistry of the prepared Fe-CDs were investigated. Transmission electron microscopy (TEM) of Fe-CDs was used to characterize the morphology and particle size distribution of the obtained Fe-CDs nanoparticles as shown in Fig. 4. The prepared Fe-CDs were well dispersed and showed a homogeneous spherical structure and no significant aggregation in water with a narrow size distribution in the range of 0.5–2.0 nm and an average particle size of 1.29 nm. Meanwhile, HR-TEM observed a lattice spacing of 0.201 nm for Fe-CDs, which belongs to the (100) interplanar spacing of graphite carbon. X-ray diffraction analysis (XRD) was used to explore the crystallinity of the prepared Fe-CDs, as shown in Fig. 5a. The experimental results showed that the prepared Fe-CDs exhibited diffraction peaks at 22.62°, 29.92°, 32.46°, 42.66°, 54.16°, 67.64°, and 71.36°, corresponding to (110), (100), (110), (111), (210), and (220), respectively. This indicates that iron has been successfully doped into the CDs, consistent with the results of Xie et al. [47]. The XRD pattern of the prepared CDs is shown in Fig. S3, where the broad peak around 22.34° corresponds to the (002) plane of graphitic carbon [20]. By analysing the Fourier transform infrared spectroscopy (FT-IR) diagrams, the forms of functional groups present on the surface of Fe-CDs can be derived. And quantitative analysis was conducted on the functional groups on the surface of Fe-CDs, and the peak area was proportional to the concentration of specific functional groups in the sample. As shown in Fig. 5b, the broad absorption from 3200 to 3400 cm^−1^ indicates the presence of hydroxyl (-OH) and amino (-NH_2_) groups in Fe-CDs [48]. Its peak area is 397.87 cm^−1^. In addition, the peaks near 1600 cm^−1^ and 1400 cm^−1^ are attributed to the C=O/C=N and -COO stretching vibrations, respectively [47,49,50]. Their peak areas are 34.25 cm^−1^ and 15.03 cm^−1^, respectively. The peak at 663 cm^−1^ is attributed to the Fe-O stretching vibrations of Fe-O-H, in agreement with the reported literature [51]. Its peak area is 34.58 cm^−1^. From the peak area, it can be inferred that Fe-CDs contain a large amount of -OH, mainly derived from solvents, with Fe-O and C=O/C=N contents being comparable [52].

**Fig. 6:**
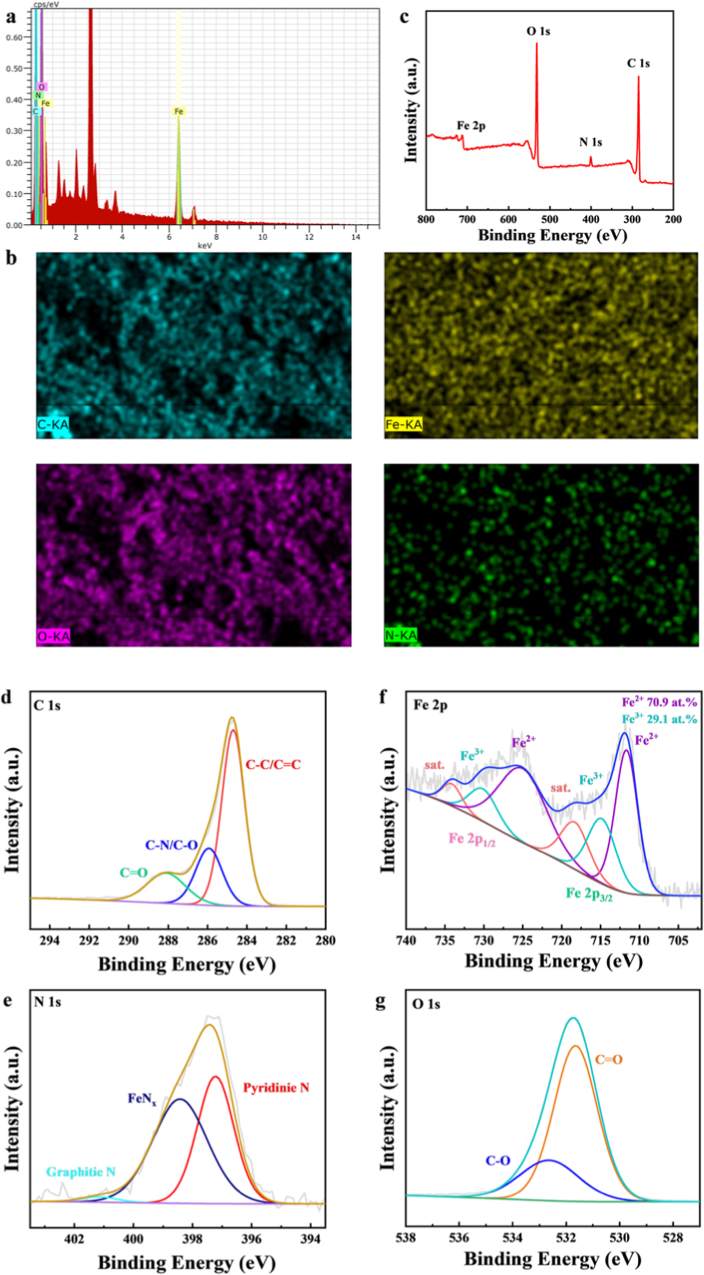
(a) EDX spectra of Fe-CDs (b) Elemental mapping of Fe-CDs (c) XPS full-range measurement spectra of Fe-CDs High-resolution XPS mapping (d) C 1s, (e) N 1s, (f) Fe 2p, (g) O 1s.

Energy Dispersive X-Ray Spectroscopy (EDX) spectra revealed the presence of four major elements, carbon, nitrogen, oxygen and iron, in the structure of Fe-CDs, as shown in Fig. 6a. The elemental mapping (EDX-MAP) indicates that these elements are uniformly distributed in the structure of Fe-CDs, as shown in Fig. 6b. In addition, the elemental analysis and chemical composition of the as prepared Fe-CDs were verified by X-ray photoelectron spectroscopy (XPS). As shown in Fig. 6c, the XPS spectrum of Fe-CDs showed four characteristic peaks, mainly carbon (C 1s, 284.8 eV), nitrogen (N 1s, 400.8 eV), oxygen (O 1s, 531.8 eV) and iron (Fe 2p, 711.80 eV), which were completely consistent with the EDX spectrum. The high-resolution C 1s spectra showed three distinct characteristic peaks at 284.7 eV, 285.9 eV and 288.1 eV, attributed to C-C/C=C, C-N/C-O, and C=O, respectively, indicating the presence of a conjugated structure [20], with a relatively high atomic percentage of carbon (67.89 %), which is indicative of a high degree of carbonation on the surface of the material [53]. The high-resolution spectrum of N 1s (Fig. 6e) shows three nitrogen characteristic peaks at 397.2 eV, 398.4 eV, and 401.2 eV, attributed to Pyridinie N, Fe-N_x_, and Graphitie N. The pyrrole nitrogen can form M-N_x_ coordination bonds to the metal, which may become the active site for catalytic reactions [20,54]. There are two pairs of peaks attributed to Fe^3+^ and Fe^2+^ in the high-resolution Fe 2p (Fig. 6f) fitting. The Fe^2+^ signals close to 711.7 eV and 725.0 eV in the Fe 2p_3/2_ orbit can be attributed to the presence of Fe-N_x_ sites, and the Fe^2+^ peak positions of Fe 2p_3/2_ are slightly shifted, because O species also participate in the coordination process, indicating that it exists in the form of Fe-O/N, which is consistent with the literature reports [55,56]. The two component peaks (714.8 eV and 730.1 eV) are attributed to Fe^3+^, peat at 718.3 eV and 734.1 eV was attributed to the overlap of vibrating satellites of iron oxide [56]. In the high-resolution O 1s spectra (Fig. 6g), there are two resolved peaks at 531.7 eV and 532.6 eV, which belong to C=O bond and C-O bond, respectively. The above data indicate the successful synthesis of Fe-CDs.

### 4.2 Peroxidase-like activity studies of Fe-CDs

**Fig. 7:**
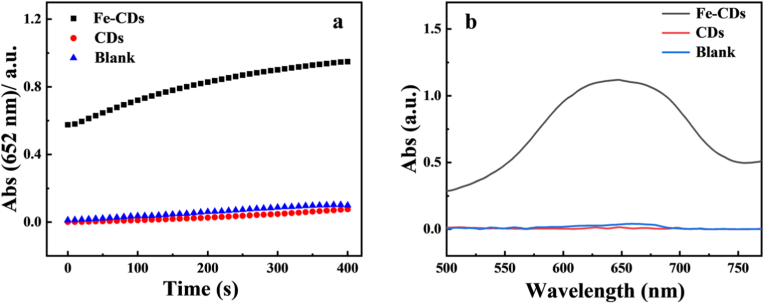
(a) Plot of oxTMB absorbance of control, CDs and Fe-CDs over time (b) Plot of oxTMB absorbance (652 nm) of control, CDs and Fe-CDs groups.

With the successful doping of Fe into CDs, we investigated the catalytic activity of the nanozyme. Colourless TMB can be oxidised to the blue product oxTMB in the presence of H_2_O_2_ and POD mimics [57], therefore, the POD-like activity of Fe-CDs and CDs on TMB was first determined, and the chemical changes in TMB were monitored by UV–Visible absorption at 652 nm. As shown in Fig. 7a, the time-dependent absorbance changes of the nanozymes Fe-CDs, CDs, and control, respectively, and the vertical coordinates represent the corresponding POD-like activities, the Fe-CDs exhibited stronger POD-like activity under the same conditions.

The activity of nanozymes depends on the reaction parameters, such as ambient temperature and time, therefore, it is necessary to explore the effects of different temperatures (4–75 °C), pH (2.5–7.2) and reaction times (5–30 min) on the catalytic efficiency of Fe-CDs and CDs (Fig. S4). The catalytic activity of Fe-CDs showed a tendency of increasing and then decreasing with increasing temperature and pH, and the optimum temperature was determined to be room temperature and the optimum pH was 3.6. Similar to most nanozymes, Fe-CDs exhibit significant catalytic activity in acidic environments and very weak catalytic activity in neutral and alkaline environments. The catalytic activity time of Fe-CDs increased intrinsically from 5 to 20 min and then began to level off, so 20 min was chosen as the optimal reaction time. In conclusion, the highest catalytic activity of Fe-CDs was achieved at pH 3.6 and room temperature for 20 min. The wavelength plots of Fe-CDs, CDs, and control at 652 nm were measured under the optimal conditions of 20 min, pH 3.6, and room temperature (Fig. 7b), and this result was in perfect agreement with the linear scanning relationship with time. The experimental results also amply demonstrated that iron doping in CDs significantly enhanced the POD-like activity, which also confirmed that the Fe element could alter the internal electronic environment and provide the active centre of CDs, thus increasing the affinity for TMB and H_2_O_2_ [20]. We conducted long-term stability and reusability evaluations on Fe-CDs. As shown in Fig. S5a, with the increase of storage days, the absorbance value slightly decreases, but then becomes stable, maintaining the stability of activity. In Fig. S5b and c, it can be seen that the enzyme activity is optimal when stored at 4 °C in the dark. In Fig. S5d, it can be observed that after three repeated uses, the relative activity can still be maintained at 96.39 %. The above experimental results indicate that the prepared Fe-CDs have good stability and reusability.

### 4.3 Steady-state kinetic analysis of Fe-CDs

**Fig. 8:**
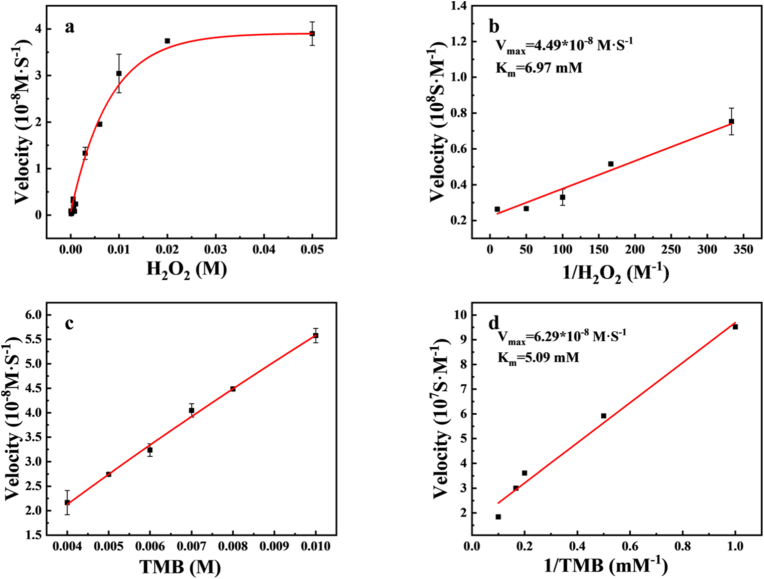
(a) Steady state kinetic determination of Fe-CDs against H_2_O_2_ (b) Double inverse plot of Fe-CDs against H_2_O_2_ (c) Steady state kinetic determination of Fe-CDs against TMB (d) Double inverse plot of Fe-CDs against TMB.

We performed steady-state kinetic analysis to systematically characterize the catalytic activity of Fe-CDs. Concentration dependent absorbance values were obtained by keeping the concentration of a substrate constant while changing the concentration of TMB or H_2_O_2_. The fitting of time and absorbance data generated the Michaelis-Menten curve, while Lineweaver-Burk of the double reciprocal plot of the Michaelis-Menten equation was used to calculate the kinetic parameters, and the double reciprocal curve equation was [58]:

$$\frac{1}{V}=\frac{Km}{Vmax}\frac{1}{\left[S\right]}+\frac{1}{Vmax}$$

where V is the velocity, V_max_ is the maximum reaction rate, [S] is the substrate concentration, and K_m_ is a constant. V_max_ and K_m_ are important parameters for determining POD-like levels [58]. When H_2_O_2_ is used as a substrate, the K_m_ value is 6.97 mM, and the V_max_ is 4.49 × 10^−8^ M s^−1^ (Fig. 8a and b). When TMB is used as a substrate, the K_m_ value is 5.09 mM, and the V_max_ is 6.29 × 10^−8^ M s^−1^ (Fig. 8c and d), by comparing the kinetic parameters of Fe-CDs with other reported nanozymes (Table S1), it can be seen that Fe-CDs have inherent peroxidase like activity [59].

### 4.4 Colourimetric detection of H_2_O_2_

**Fig. 9:**
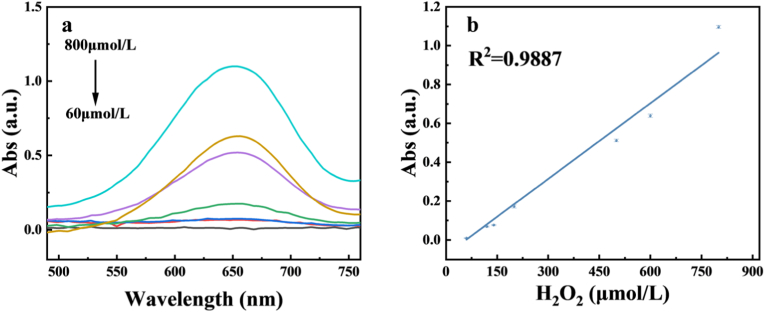
(a) Variation of absorbance at 652 nm as a function of H_2_O_2_ concentration (b) Linear plot for detection of H_2_O_2_ concentration.

Given the POD-like activity of Fe-CDs, with which we can detect H_2_O_2_ colorimetrically, the degree of oxidation of TMB is dependent on the amount of H_2_O_2_, and therefore a concentration range of 60–800 μΜ was investigated. As shown in Fig. 9a and b, the absorbance value at 652 nm increased gradually with the increase of H_2_O_2_ concentration, and further experiments revealed that the concentration and absorbance value showed a linear relationship, with a linear equation of y = 0.0013x-0.07575, a correlation coefficient of R^2^ = 0.9887, and a limit of detection of LOD = 7.25 μM (LOD = 3σ/s). In the range of certain concentrations, the detection limit of our prepared Fe-CDs was significantly better than that of other nanozymes (Table 1). Therefore, it provides a convenient and efficient colourimetric method for the detection of H_2_O_2_.

**Table 1:** Comparison of prepared Fe-CDs with other nanozymes.

Catalyst	Substrate	Linear range	Detection limit	Detecting substance	Ref.
Fe_3_O_4_@Cu@Cu_2_O	TMB	4–50 mM	2 mM	H_2_O_2_	[55]
NiCo_2_S_4_@N,S-rGO	TMB	0.04–50 mM	12 μM	H_2_O_2_	[56]
Au@Pt	OPD	45–1000 μM	45 μM	H_2_O_2_	[57]
MMT-CuS	TMB	30–200 μM	24.7 μM	H_2_O_2_	[58]
FePt/GO	TMB	30–500 μM	22 μM	H_2_O_2_	[59]
GBR	TMB	0.5–5 mM	417 μM	H_2_O_2_	[60]
[Pyr]Ac- Ni^0^	TMB	400–4000 μM	120 μM	H_2_O_2_	[61]
Fe_3_O_4_ -GBR	TMB	100–880 μM	49.6 μM	H_2_O_2_	[62]
Fe-CDs	TMB	60–800 μM	7.25 μM	H_2_O_2_	This work

### 4.5 Specific detection of H_2_O_2_

**Fig. 10:**
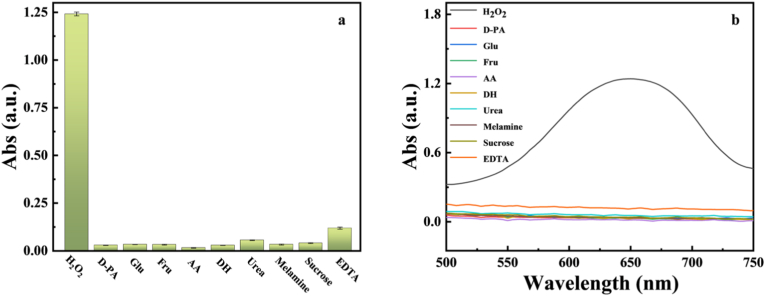
Colourimetric detection of molecular interferences (a) Histogram (b) Wavelength plot.

**Fig. 11:**
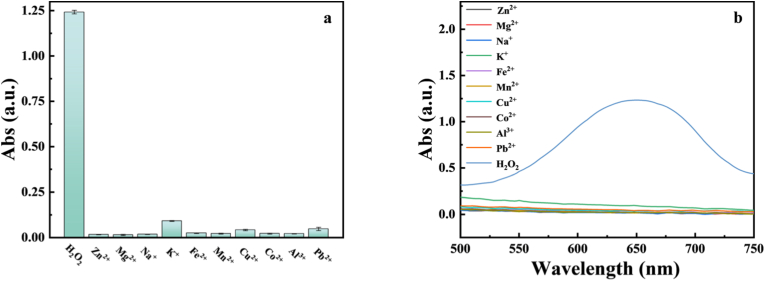
Colourimetric detection of ionic interferences (a) Histogram (b) Wavelength plot.

Selective determination, as an important evaluation factor, was monitored by introducing potentially interfering substances into the Fe-CDs/TMB system for specific colourimetric detection, using UV–Visible absorption, as shown in Fig. 10, Fig. 11. We selected 9 kinds of molecular substances (D-PA, Glu, Fru, AA, DH, Urea, Melamine, Sucrose and EDTA) and 10 kinds of common metal ion solutions (Zn^2+^, Mg^2+^, Na^+^, K^+^, Fe^2+^, Mn^2+^, Cu^2+^, Co^2+^, Al^3+^ and Pb^2+^) and added these interfering substances into the system, and diluted them with ultrapure water to a fixed volume. It was found that the interferents, whether molecular substances or ionic solutions, had no obvious absorption peak at 652 nm, while only H_2_O_2_ had obvious absorption characteristic peak. The above results show that the colorimetric method has good selectivity for H_2_O_2_.

### 4.6 Actual sample testing

In order to demonstrate the practical applicability of the sensing platform by applying the method to real samples to detect H_2_O_2_, we diluted purchased mouthwash. Triplicate recovery experiments (600 μM, 700 μM and 800 μM) were performed by adding different concentrations of H_2_O_2_ to inject the Fe-CDs/TMB system containing mouthwash under optimised conditions, and the results are listed in Table 2. The recoveries of H_2_O_2_ in mouthwash ranged from 98.495 % to 104.01 % with RSDs from 1.8 % to 5.2 %. Similarly, laboratory tap water was used for spiked detection. By injecting 80 μM, 90 μM, and 140 μM H_2_O_2_ into the Fe-CDs/TMB system containing tap water, the results are shown in Table 3. The recovery rate of H_2_O_2_ in tap water was 96.39 %–102.72 %, with RSDs ranging from 0.59 % to 2.8 %, these results are in agreement with those obtained in the previous experiments. The Fe-CDs/TMB colourimetric system we have developed simplifies and reduces the cost of analysis and can be used for the determination of H_2_O_2_ in real samples.

**Table 2:** Detection of H_2_O_2_ in mouthwash.

Sample	Added (μM)	Found (μM)	Recovery (%)	RSD (%)
Mouthwash	600	624.06	104.01	5.2
	700	701.41	100.20	2.8
	800	787.96	98.495	1.8

**Table 3:** Detection of H_2_O_2_ in Laboratory tap water.

Sample	Added (μM)	Found (μM)	Recovery (%)	RSD (%)
Laboratory tap water	80	80.37	100.46	1.9
	90	92.45	102.72	0.59
	140	134.94	96.39	2.8

### 4.7 AND logic gate construction

**Fig. 12:**
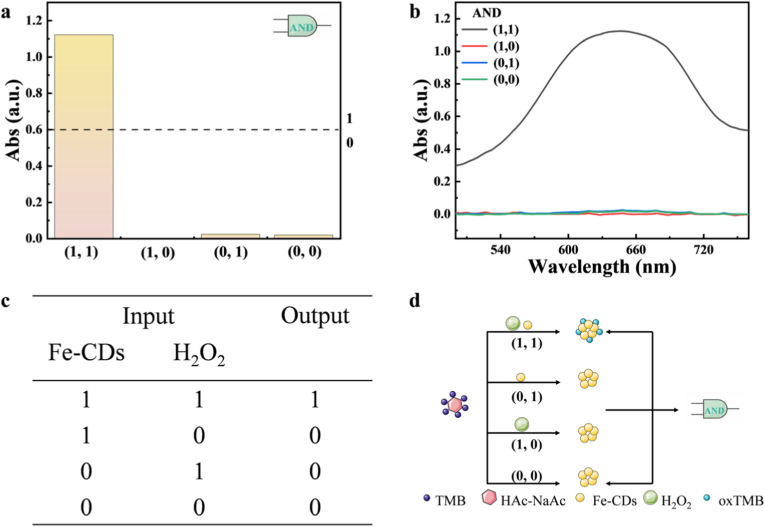
(a) Histogram of AND logic gate with Fe-CDs and H_2_O_2_ as inputs and absorbance value as output (b) Spectrogram of AND logic gate with Fe-CDs and H_2_O_2_ as inputs and absorbance value as output (c) Schematic representation of different combinations of inputs and outputs (d) Schematic representation of AND logic gate with Fe-CDs and H_2_O_2_ as inputs.

AND logic gate is one of the more widely used dual-input logic gates, whose logical connotation is that the output is a high signal “1” when and only when “1” is input at the same time, otherwise the output is a low signal “0” [21]. Using the POD-like activity of Fe-CDs, TMB can be oxidised to blue oxTMB to construct AND logic gate under optimal conditions. An AND logic gate for H_2_O_2_ colourimetric sensing was constructed in the HAc-NaAc system with TMB as the colour developer. As shown in Fig. 12, Fe-CDs and H_2_O_2_ were used as inputs 1 and 2, respectively, and whether or not the TMB was oxidised to blue oxTMB was used as the output signal. As shown in Fig. 12a, where the absorbance value of 0.6 is used as the colourimetric threshold, when Fe-CDs and H_2_O_2_ are not added to this system, i.e., (0, 0), at this time, TMB cannot be oxidised, the absorbance value is very small, and the output is a low signal “0”. Adding only Fe-CDs, i.e., (1, 0), or only H_2_O_2_, i.e., (0, 1), to this system, the absorbance values were still very low and the output signals were recorded as “0”. And when the input is (1, 1), the added Fe-CDs and H_2_O_2_ can oxidise the TMB to a blue product, noting this highly colorimetric signal output as “1”. Thus by controlling different combinations of inputs from Fe-CDs and H_2_O_2_, an AND gate operating logic can be implemented and this logic platform can be used for colourimetric sensing of H_2_O_2_.

### 4.8 Evaluation of antimicrobial capacity of Fe-CDs

Bacterial infection is a key factor in slowing or significantly delaying wound healing, and H_2_O_2_, a low-cost traditional antiseptic, is a ROS species commonly used in clinical medicine to remove bacteria. In pharmaceutical and healthcare environments, high concentrations of H_2_O_2_ are necessary to achieve the desired disinfection efficiency, but high concentrations of H_2_O_2_ are harmful to human health and may delay wound healing. It was found that the POD-like catalytic activity of Fe-CDs was utilised to convert low concentrations of H_2_O_2_ into ·OH, which has a higher oxidative activity than H_2_O_2_, thus killing bacteria and effectively solving the problem of antibacterial activity of low concentrations of H_2_O_2_.

**Fig. 13:**
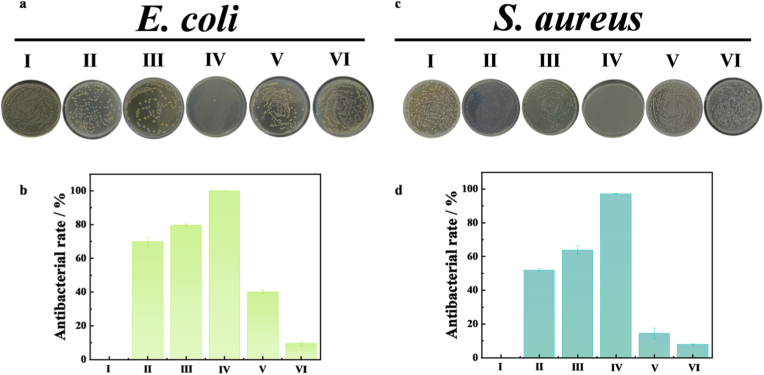
(a) Digital photographs of *E. coli* and (c) *S. aureus* colony agar plates (b) Histograms of *E. coli* and (d) *S. aureus* antibacterial Rate assays.

In this experiment, we chose *E. coli* and *S. aureus* to evaluate the antimicrobial effects of Fe-CDs and CDs, and the results are shown in Fig. 13. In Fig. 13a and b, we tested the antimicrobial capacity in *E. coli*, the first three groups are the control, and the fourth and fifth groups are the antimicrobial effect of adding Fe-CDs and CDs, respectively, the sixth group consists only of CDs, and it can be seen that the antimicrobial efficiency of the CDs doped with elemental iron can be as high as 99.89 %, while the antimicrobial efficiency of the CDs alone is 7.89 %. Similarly, we did six sets of experiments on *S. aureus*, as shown in Fig. 13c and d, and the results of the experiments were similar, with the antimicrobial capacity of Fe-CDs (97 %) being much stronger than the antimicrobial capacity of CDs (8.1 %). This phenomenon further provides ample evidence that Fe-CDs can efficiently catalyse H_2_O_2_ to produce ·OH with high oxidative capacity, which in turn causes oxidative damage to bacteria. For *E. coli* and *S. aureus*, their antibacterial effects differ under the same conditions, with *E. coli* exhibiting superior antibacterial activity. This difference comes from the different structures of the cell membrane and cell wall in *E. coli* and *S. aureus* [63]. The main cause of bacterial death or damage is the leakage of cytoplasmic contents due to damage to the bacterial cell wall. The surface of *E. coli* is negatively charged due to the presence of lipopolysaccharides in the cell membrane, while the zeta potential of Fe-CDs is 2.29 mV (Fig. S6). Positive and negative charge attraction makes Fe-CDs easily bind to the bacterial membrane, thereby damaging the cell wall. Compared with *E. coli*, *S. aureus* has a thicker cell wall, so its damage to *E. coli* is greater because they lack a peptidoglycan layer as a metal protective layer [63].

## 5 Conclusion

In summary, we synthesised Fe-CDs with POD-like activity by a one-step hydrothermal method using Scutellaria baicalensis dregs for colorimetric sensing of H_2_O_2_ and safe and effective antimicrobial therapy. The experimental results showed that Fe-CDs exhibited significantly enhanced POD-like activity compared to pure CDs, and steady-state kinetic analysis also confirmed the POD-like activity of Fe-CDs. Using the POD-like activity of Fe-CDs that can oxidise TMB to blue oxTMB, an AND logic platform was constructed for colourimetric sensing of H_2_O_2_ and can be used for the detection of real samples. It was illustrated by TEM images that the prepared Fe-CDs had a particle size of 1.29 nm, and their nanoscale size facilitated their entry into bacteria for excellent antimicrobial properties. In vitro antibacterial assay showed that the inhibition rate of Fe-CDs was 99.89 % for *E. coli* and 97 % for *S. aureus*, while the antibacterial rate of CDs was 7.89 % and 8.1 % for *E. coli* and *S. aureus*, respectively. The antibacterial effect of Fe-CDs was stronger than that of CDs, which also fully proved that the POD-like activity of Fe-CDs was stronger than that of CDs. This work synthesised a green and safe antimicrobial nanozymes based on herbal dregs, which solved the problem of herbal dregs disposal, and constructed a logical platform using colorimetric signals as outputs, the colorimetric coupling method simplified the analysis process and reduced the cost, and also provided promising insights into the development of novel antimicrobial materials. The current Fe-CDs may still have catalytic activity that needs to be improved and stability issues in complex practical samples. In the future, these limitations can be further addressed by surface modification and functionalization, constructing composite materials, or regulating the morphology of iron species. The precise elucidation of Fe-CDs penetration pathways and microscopic morphological changes in bacteria requires further exploration through more specialized techniques in the future. Further exploration can be conducted on more efficient and convenient synthesis methods to synthesise heteroatom doped CDs, and a multifunctional sensing platform can be constructed, such as developing a supporting mobile APP that captures color changes using a mobile camera for quantitative analysis, making detection more household oriented.

## CRediT authorship contribution statement

**Xiangru Hou:** Writing – original draft, Formal analysis, Data curation, Conceptualization. **Denggerile Ao:** Software, Resources, Project administration. **Lu Ga:** Supervision, Project administration, Funding acquisition. **Gang Dai:** Software, Project administration, Methodology. **Jun Ai:** Writing – review & editing, Project administration, Funding acquisition.

## Formula

1. Antibacterial ratio% = (1 − $\frac{{CFU}_{\left(each\;group\right)}}{{CFU}_{\left(control\right)}}$) × 100 %
2 $\frac{1}{V}=\frac{Km}{Vmax}\frac{1}{\left[S\right]}+\frac{1}{Vmax}$
3 LOD = 3σ/s

## Declarations

Ethics approval and consent to participate Not applicable.

## Consent for publication

All authors agree to publish.

## Funding

The National 10.13039/501100001809Natural Science Foundation of China (Grant No. 21864020), Science and technology planning project of Inner Mongolia Autonomous Region: 2021GG0367.

## Declaration of competing interest

The authors declare that they have no known competing financial interests or personal relationships that could have appeared to influence the work reported in this paper.

## Data Availability

All data and materials in this study are included in the published article and its additional file.
