# Supplementary material for: Nature’s chemical computer: Herbal residue-derived carbon dots build logic gates for H2O2 tracking and ecological antimicrobials
Source: Mater Today Bio. 2025 Nov 20;35:102574. doi: 10.1016/j.mtbio.2025.102574 (PMC12702416; doi:10.1016/j.mtbio.2025.102574)
Supplement: Multimedia component 1 [file mmc1.docx]

**Supporting Information**

**Nature's chemical computer: herbal residue-derived carbon dots build logic gates for H_2_O_2_ tracking and ecological antimicrobials**

Xiangru Hou^a#^, Denggerile Ao ^a#^, Lu Ga^b*^, Gang Dai ^a*^, Jun Ai^a*^

a.College of Chemistry and Enviromental Science, Inner Mongolia Key Laboratory of Environmental Chemistry, Inner Mongolia Normal University, 81 zhaowudalu, Hohhot 010022, China

b.College of Pharmacy, Inner Mongolia Medical University, Jinchuankaifaqu, Hohhot,010110, China

*Corresponding author’s E-mail: [imacaj01@163.com](mailto:imacaj01@163.com).

^#^Xiangru Hou and Denggerile Ao contributed equally to this work.

**Contents**

1. Fluorescence spectra and UV-Vis absorption spectra of CDs………… Fig. S1
2. Stability diagrams of CDs……………………………………………... Fig. S2
3. XRD image of CDs……………………………………………………. Fig. S3
4. Optimisation plot of peroxidase-like activity of Fe-CDs……………… Fig. S4
5. Long term stability and reusability evaluation of Fe-CDs…………….. Fig. S5
6. Comparison of steady-state kinetics of prepared Fe-CDs with other nanozymes …………………………………………………………… Table. S1
7. Zeta potential of Fe-CDs………………………………………………. Fig. S6


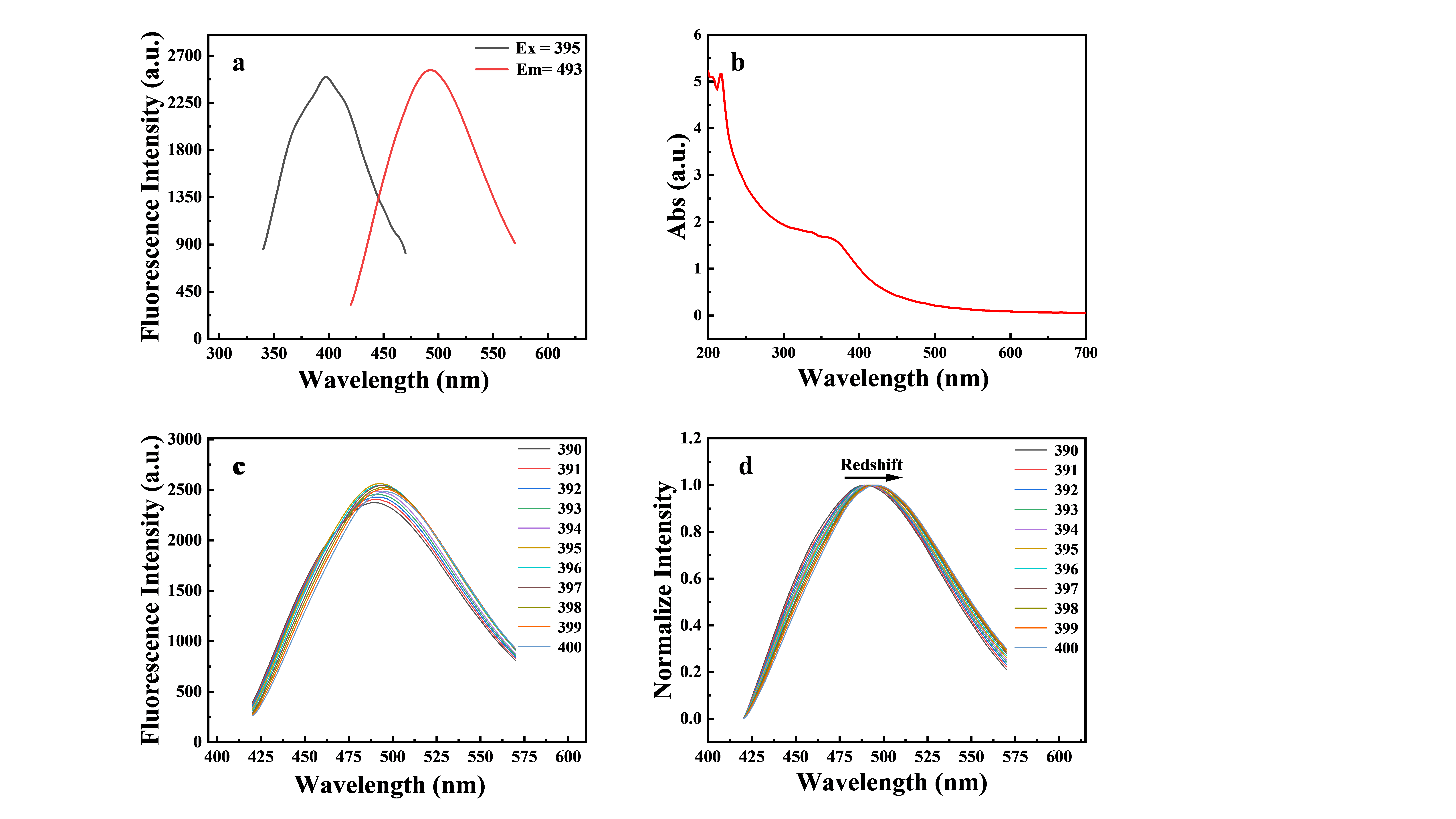


**Fig. S1**(a) Fluorescence spectra of CDs (b) UV-Vis absorption spectra of CDs (c) Emission spectra at different excitation wavelengths (d) Normalized spectrum.


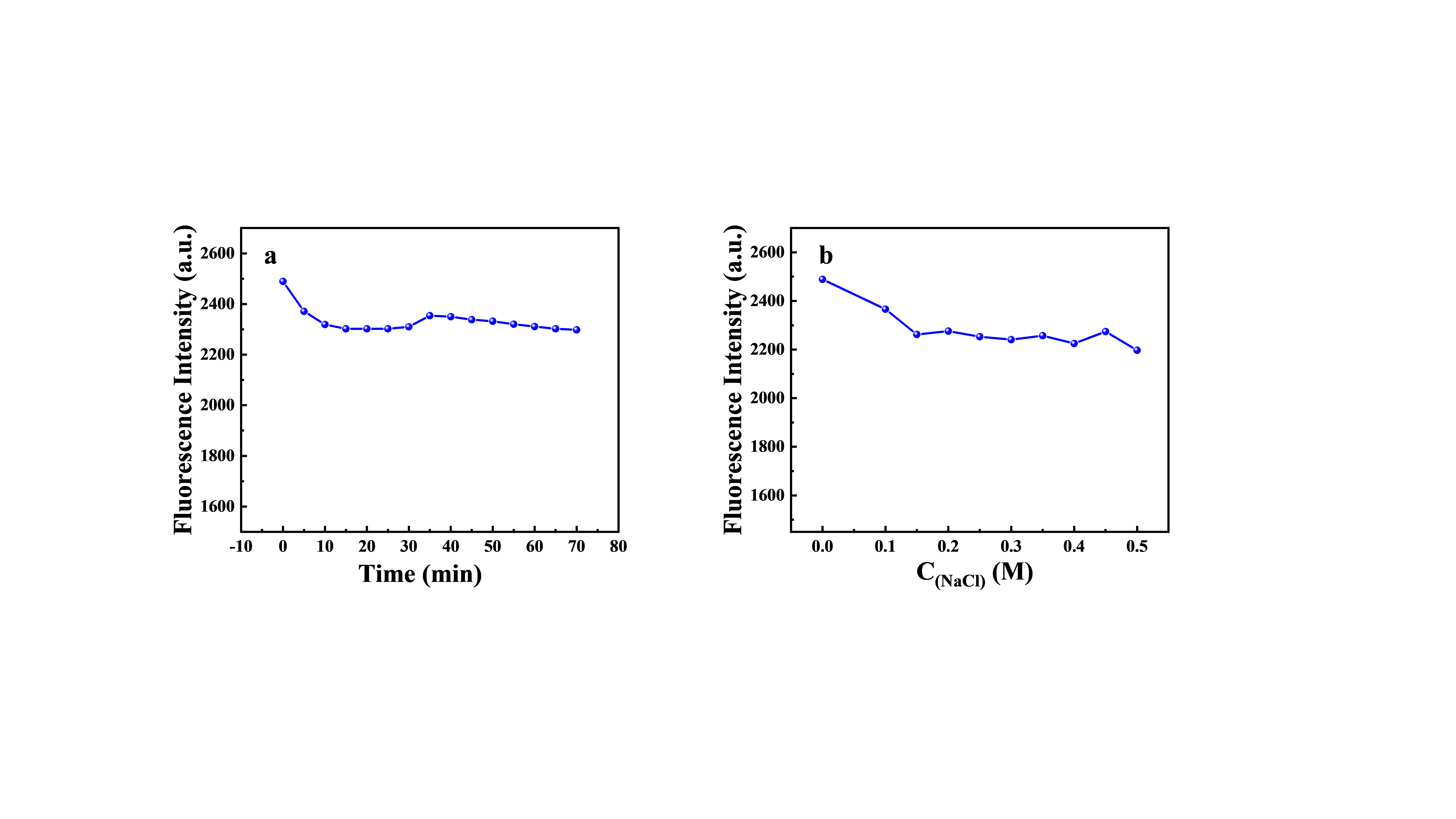


Fig.S2 (a) CDs photobleaching fluorescence scatter diagram (b) Fluorescence scatter diagram of the effect of different concentrations of NaCl solution on CDs.


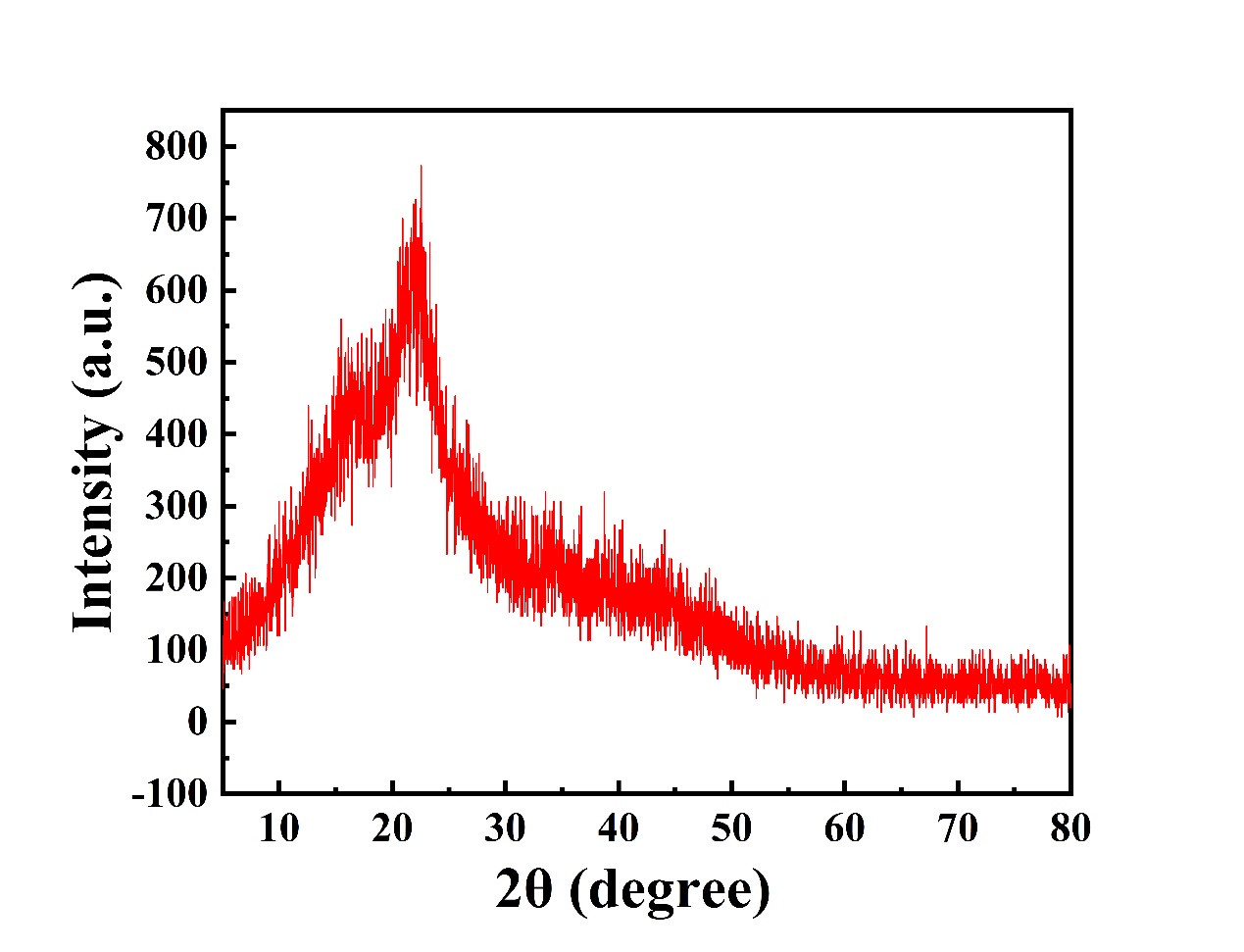


Fig.S3 XRD image of CDs.


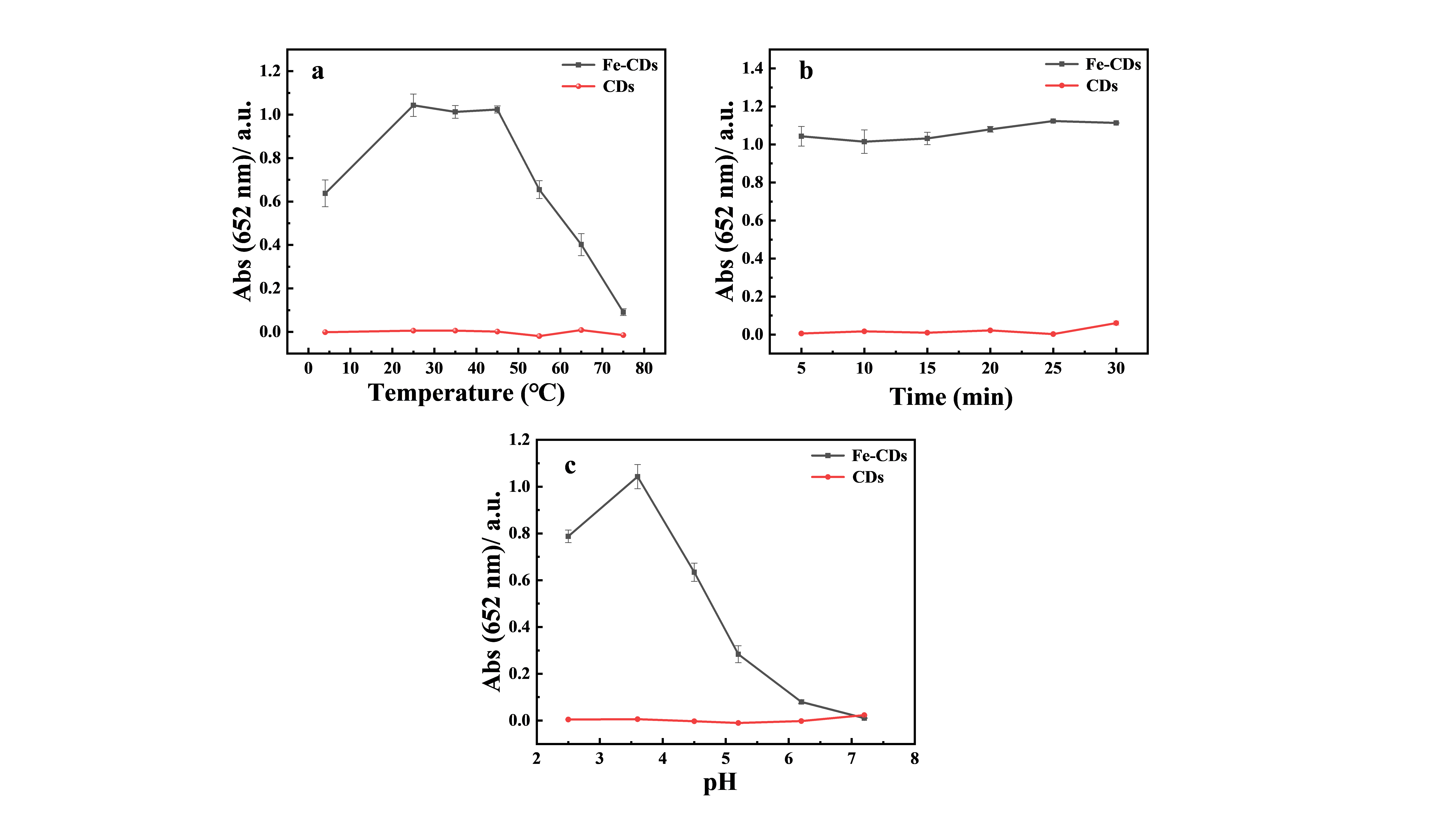


Fig.S4 Effect of peroxidase like activity of Fe-CDs and CDs on (a) temperature (b) reaction time (c) pH (error bars in the figure are three parallel experimental values).


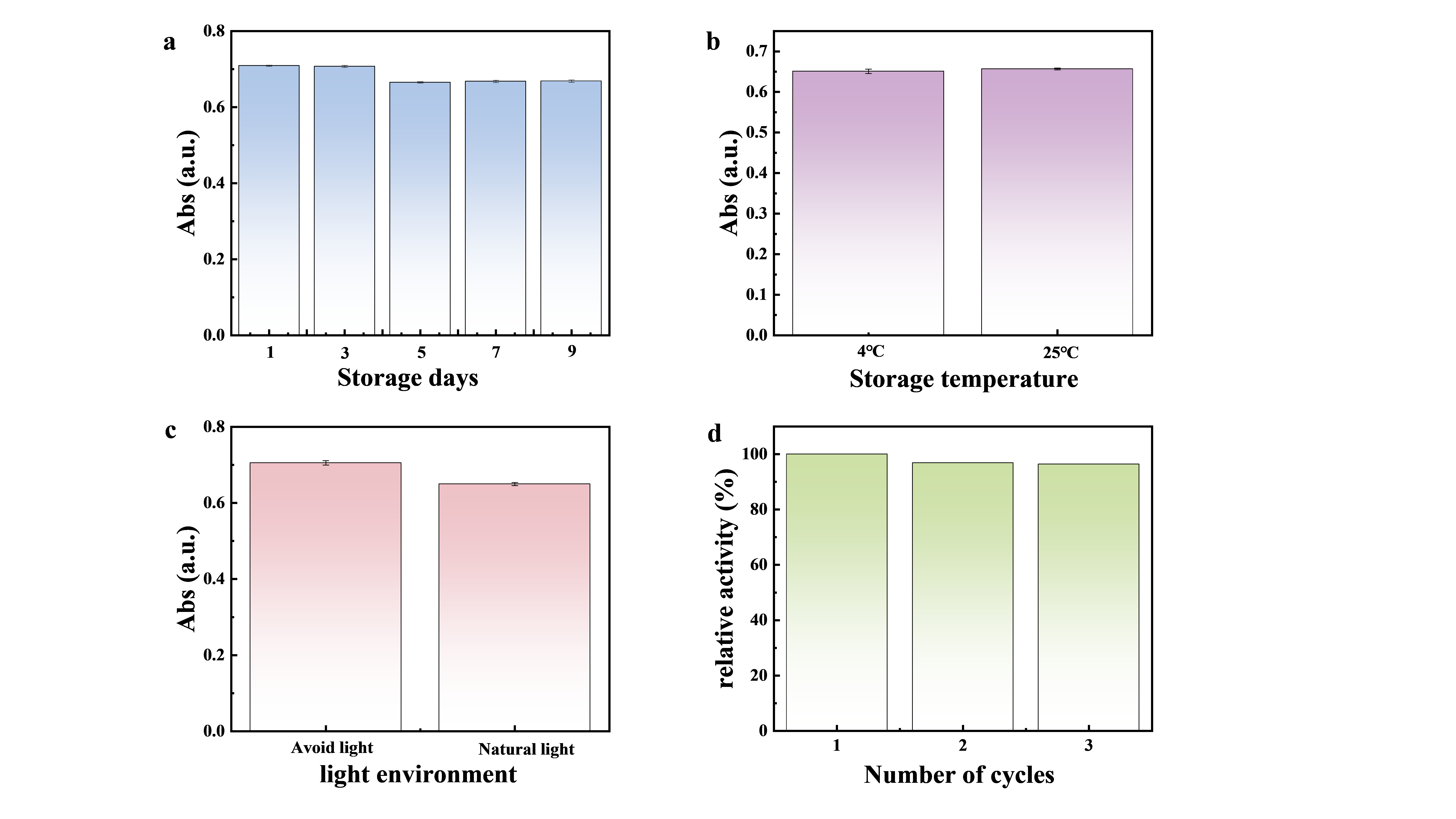


Fig.S5 Long term stability and reusability evaluation of Fe-CDs (a) storage days (b) storage temperature (c) light environment (d) reusable frequency (error bars in the figure are three parallel experimental values).

Table S1 Comparison of steady-state kinetics of prepared Fe-CDs with other nanozymes.

| **Catalyst** | **Substrate** | **K_m_ (mM)** | **V_max_ (10^-8^M·S^-1^)** | **Reference** |
| --- | --- | --- | --- | --- |
| PB/g-C_3_N_4_ | TMB  H_2_O_2_ | 0.424  76.7 | 17  34 | [1] |
| GBR | TMB  H_2_O_2_ | 0.83  10.98 | 0.68  3.60 | [2] |
| Fe_3_O_4_ | TMB | 0.098 | 3.44 | [3] |
|  | H_2_O_2_ | 154 | 9.78 |  |
| Fe-CDs | TMB | 5.09 | 6.29 | This work |
|  | H_2_O_2_ | 4.82 | 4.49 |  |

[1] Zhou D, Wang C, Luo J, et al. C_3_N_4_ nanosheet-supported Prussian Blue nanoparticles as a peroxidase mimic: colorimetric enzymatic determination of lactate[J]. Microchimica Acta, 2019, 186(11): 735.

[2] Singh S, Mitra K, Singh R, et al. Colorimetric detection of hydrogen peroxide and glucose using brominated graphene[J]. Analytical Methods, 2017, 9(47): 6675-6681.

[3] Liu Y, Xu B, Lu M, et al. Ultrasmall Fe-doped carbon dots nanozymes for photoenhanced antibacterial therapy and wound healing[J]. Bioactive materials, 2022, 12: 246-256.


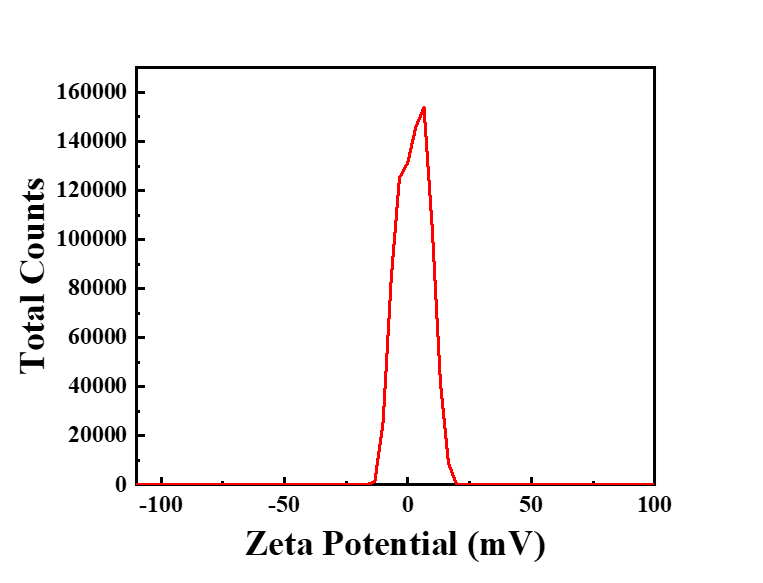


Fig.S6 Zeta potential of Fe-CDs.
